# Long non-coding RNAs play an important regulatory role in tumorigenesis and tumor progression through aerobic glycolysis

**DOI:** 10.3389/fmolb.2022.941653

**Published:** 2022-08-22

**Authors:** Ni Fan, Hui Fu, Xuchen Feng, Yatong Chen, Jingyu Wang, Yuqi Wu, Yuhong Bian, Yingpeng Li

**Affiliations:** ^1^ College of Chinese Materia Medica, Tianjin University of Traditional Chinese Medicine, Tianjin, China; ^2^ College of Integrated Chinese and Western Medicine, Tianjin University of Traditional Chinese Medicine, Tianjin, China; ^3^ Engineering Research Center of Modern Chinese Medicine Discovery and Preparation Technique, Ministry of Education, Tianjin University of Traditional Chinese Medicine, Tianjin, China

**Keywords:** lncRNAs, aerobic glycolysis, tumorigenesis, tumor progression, enzyme

## Abstract

Compared to normal cells, cancer cells generate ATP mainly through aerobic glycolysis, which promotes tumorigenesis and tumor progression. Long non-coding RNAs (LncRNAs) are a class of transcripts longer than 200 nucleotides with little or without evident protein-encoding function. LncRNAs are involved in the ten hallmarks of cancer, interestingly, they are also closely associated with aerobic glycolysis. However, the mechanism of this process is non-transparent to date. Demonstrating the mechanism of lncRNAs regulating tumorigenesis and tumor progression through aerobic glycolysis is particularly critical for cancer therapy, and may provide novel therapeutic targets or strategies in cancer treatment. In this review, we discuss the role of lncRNAs and aerobic glycolysis in tumorigenesis and tumor progression, and further explore their interaction, in hope to provide a novel therapeutic target for cancer treatment.

## 1 Introduction

In recent years, the incidences of tumors have been increasing dramatically, the morbidity rates are increasing in the younger population (Sung et al., 2021). Cancer has become one of the leading causes of death in the human population (Feng et al., 2019; Mattiuzzi and Lippi, 2020). However, the occurrence of cancer involves a complex biological mechanism (Ping et al., 2021), and one of the critical mechanisms of cancer is altered metabolism (Shuvalov et al., 2021), which is cancer metabolic reprogramming. It provides tumor cells with the energy and structural resources necessary for excessive cell proliferation and growth, and has been widely regulated by activation of oncogenes or loss of tumor suppressors (Liu et al., 2019). The uniquely reprogrammed metabolic phenotype exhibited by tumor cells known as the Warburg effect or aerobic glycolysis, which is characterized by high rates of aerobic glycolysis, leads to the production of lactic acid and reduced mitochondrial oxidative phosphorylation even in the presence of oxygen (Warburg, 1956; Liberti and Locasale, 2016). Some signaling pathways play important roles in glucose metabolism, such as PI3K/Akt/mTOR pathway, JAK/STAT pathway, P53 pathway, and so on.

Long non-coding RNAs (LncRNAs) are a heterogeneous group of transcripts of more than 200 nucleotides in length. They regulate numerous cellular processes, primarily through physical interaction with other molecules. LncRNAs are also involved in the ten hallmarks of cancer, including enabling replicative immortality (Chu et al., 2017), sustaining proliferative signaling (Wang et al., 2018b), evading growth suppressors (Hu et al., 2019), resisting cell death (Zhang et al., 2020b), tissue invasion and metastasis (Hanniford et al., 2020), inducing angiogenesis (Wang et al., 2020b), genome instability and mutation (Elguindy and Mendell, 2021), tumor-promoting inflammation (Ahmad et al., 2021), deregulating cell energetics (Chen et al., 2019b), avoiding immune destruction (Jiang et al., 2017). Multiple studies have documented an aberrant lncRNA expression in various cancers where they act as oncogenes or tumor suppressors (Fu et al., 2019; Barth et al., 2020).

Some lncRNAs contributed to cancers are involved in metabolic alterations (Balihodzic et al., 2021). LncRNAs-derived metabolism reprogramming allows cancer cells to maintain deregulated proliferation and withstand hostile microenvironments such as energy stress (Liu et al., 2019), and the probable mechanism of this is that lncRNAs can upregulate metabolic enzymes, disturb metabolic signaling pathways, and modulate the expression of oncogenic or tumor-suppressive genes (Balihodzic et al., 2021). Additionally, lncRNAs are also increasingly being considered potential therapeutic targets (Chandra Gupta and Nandan Tripathi, 2017). Thus, the interaction between lncRNAs and metabolic reprogramming, especially aerobic glycolysis needs further studies. In this review, we mainly discuss the connection between lncRNAs, aerobic glycolysis, and cancer, primarily illustrating the mechanism of lncRNAs regulating tumorigenesis and tumor progression through aerobic glycolysis.

## 2 Long non-coding RNAs play an important regulatory role in tumorigenesis and tumor progression

**FIGURE 1:**
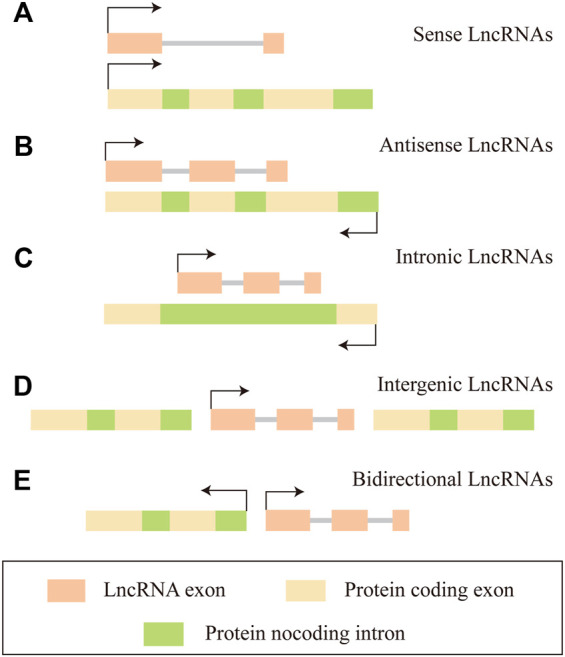
The classification of Long non-coding RNAs (LncRNAs). **(A)** Sense LncRNAs, it is transcribed by sense chain of protein coding gene, overlaps with at least one exon of protein coding gene on the same chain. **(B)** Antisense LncRNAs, it is transcribed by the complementary DNA strand of protein coding gene, overlaps with at least one exon of the positive gene. **(C)** Intronic LncRNAs, it is located in the intron region of a protein coding gene without overlapping with its exons. **(D)** Intergenic LncRNAs, it is located between two protein coding genes and can be transcribed independently. **(E)** Bidirectional LncRNAs, they share the same promoter with protein coding genes, but the transcription direction is opposite to protein coding genes.

**FIGURE 2:**
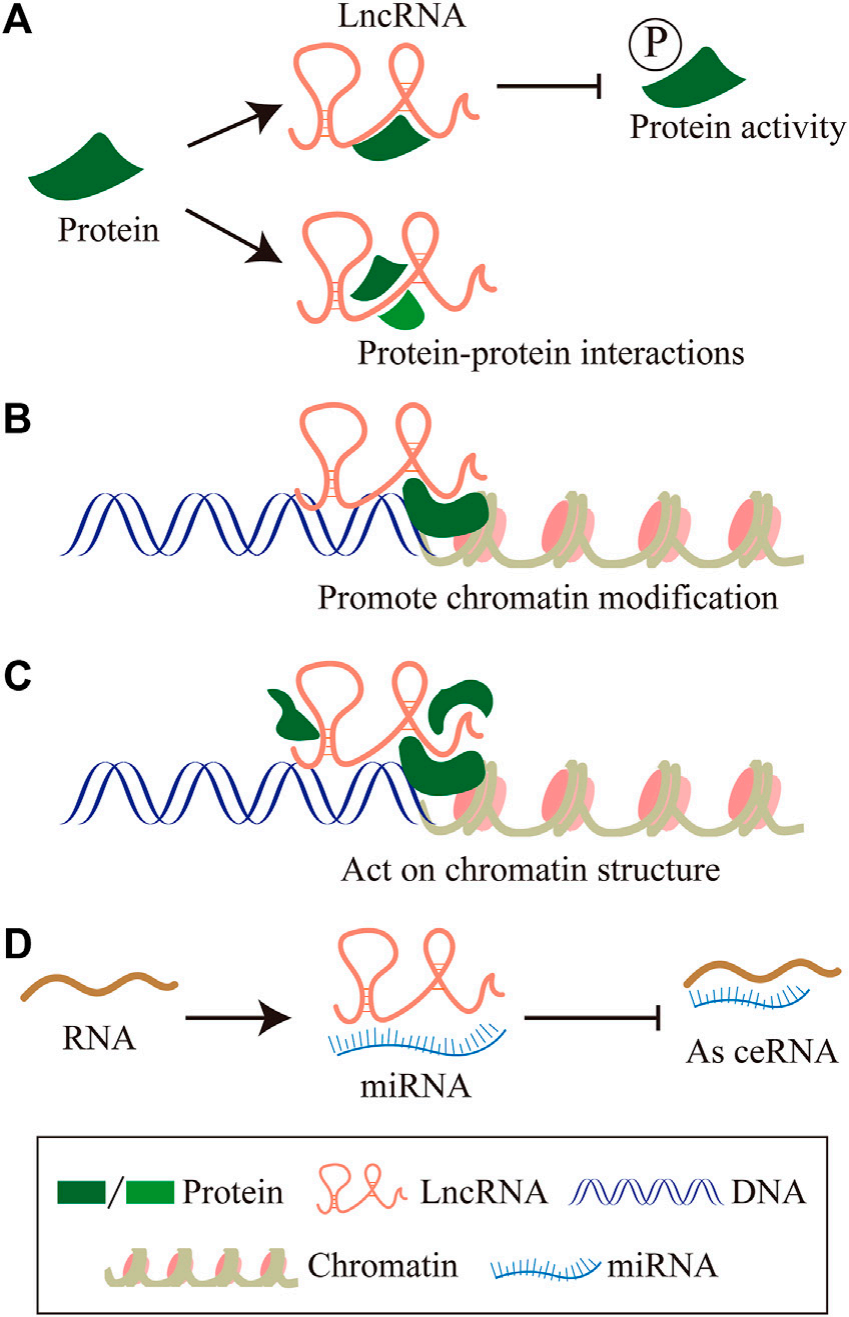
The molecular mechanism of LncRNAs. **(A)** LncRNAs interact with specific protein to participate in global cellular processes by regulating protein activity or modulating protein-protein interactions as scaffolds to facilitating the formation of protein complex. **(B)** LncRNAs recruit chromatin modification complexes to the promoter region of chromatin, and promote chromatin modification. **(C)** LncRNAs recruit the DNA of RNA binding proteins (RBPs), remodel chromatin structure, thus regulating the expression of target genes. **(D)** LncRNAs sequester miRNAs from target mRNAs as competitive endogenous RNA.

About 75% of the human genome can be transcribed into RNA, less than 2% of this group encode proteins, and the vast majority of transcripts are non-coding RNAs (ncRNAs) (2012). The proportion of ncRNAs in the human genome is much lower than that in low-level organisms, which suggests the importance of non-coding RNAs in biological evolution (Birney et al., 2007; ENCODE Project Consortium, 2012). NcRNAs can be divided into two main categories according to the molecular structure, linear RNA and circular RNA. The linear RNAs are further classified as short non-coding RNAs (ncRNAs) and long non-coding RNAs (lncRNAs). LncRNAs are composed of six types according to the genomic location: sense lncRNA, antisense lncRNA, intronic lncRNA, intergenic lncRNA, and bidirectional lncRNA (Zhang et al., 2020c) (Figure 1). In summary, lncRNAs regulating gene expressions at multiple levels, are defined as a group of transcripts longer than 200 nucleotides with little or without evident protein-encoding function (Batista and Chang, 2013). In the past decades, lncRNAs have gradually become one of the most popular molecules in biomedical research. Recent studies have shown that lncRNAs are associated with many important physiologic and pathologic processes, including differentiation, development, and disease. The molecular mechanism involved has been concluded as follows (Huang et al., 2020; Statello et al., 2021): 1) LncRNAs interact with specific protein to participate in global cellular processes by regulating protein activity or modulating protein-protein interactions as scaffolds to facilitating the formation of the protein complex. 2) LncRNAs recruit chromatin modification complexes to the promoter region of chromatin and promote chromatin modification. 3) LncRNAs recruit the DNA of RNA binding proteins (RBPs) and remodel chromatin structure, thus regulating the expression of target genes. 4) LncRNAs sequester miRNAs from target mRNAs as competitive endogenous RNA (Figure 2).

LncRNAs have been determined to be involved in regulating a variety of physiological processes during tumor development, including proliferation, apoptosis, metastasis, maintaining the stemness property of cancer stem cells (CSCs), tumor-related inflammation, etc. For example, LINC00926 inhibits breast cancer cell proliferation, invasion, and metastasis both *in vivo* and vitro (Chu et al., 2021). LncRNA HOTAIR functions as a ceRNA for tumor-suppressive miRNAs to induce the CSC phenotype in hepatocellular carcinoma (HCC) under hypoxia (Hu et al., 2020). LncRNA GNAS-AS1 redirected the polarization of macrophages in tumor microenvironment and promoted the migration and invasion of non-small cell lung cancer (Li et al., 2020c). LncRNA FENDRR potentiates tumorigenicity and cell growth in hepatocellular carcinoma mainly through suppressing Treg-mediated immune evasion of cancer cells by competitively bounding to miR-423-5p (Yu et al., 2019). LncRNA GAS5 could enhance the killing effect of NK cells on liver cancer by sponging miR-544 to target RUNX3, which augments NK cell cytotoxicity, IFN-γ secretion, and the proportion of CD107a^+^ NK cells (Fang et al., 2019). Therefore, lncRNAs are promising targets in tumors because of their important roles in tumorigenesis and tumor progression.

Unfortunately, even though more than 40,000 lncRNAs have been found in human tissues, statistics from Human GENCODE suggest that the human genome contains more than 16,000 lncRNA genes (Fang et al., 2018; Uszczynska-Ratajczak et al., 2018), however, most of them have not been reported in the literature. Until now, the molecular mechanisms of lncRNAs in regulating biological processes are unknown to a large extent. Exploring the role of lncRNAs in eukaryotic cells, especially in cancer cells may reveal new rules and mechanisms for regulating physiological processes. It will annotate and clarify the structure and function of the genome from a different perspective from protein-coding genes, and analyze the essence of life activity more comprehensively.

## 3 Aerobic glycolysis is closely associated with tumorigenesis and tumor progression

**FIGURE 3:**
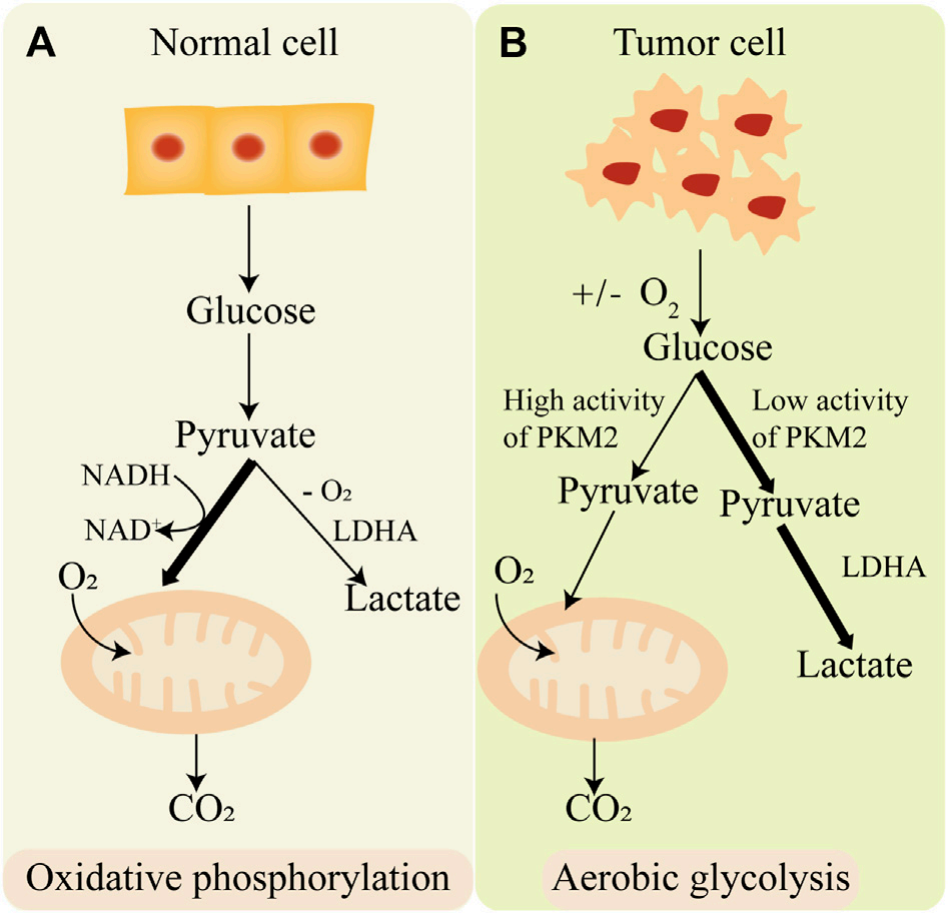
The difference of oxidative phosphorylation and aerobic glycolysis. **(A)** Normal cell produces energy mainly through oxidative phosphorylation. **(B)** Cancer cell produces energy mainly through aerobic glycolysis.

Glycolysis is an important process of cell glucose metabolism, during the process glucose degrades into acetone acid and synthesis ATP in cytoplasm (Gatenby and Gillies, 2004). Glycolysis is the initial enzymatic decomposition reaction of glucose in eukaryotic cells and bacteria. It has important physiological significance for cells in hypoxia and some special physiological and pathological conditions. In the 1920s, Warburg found that tumor cells employ aerobic glycolysis coupling with reduced mitochondrial oxidative phosphorylation for energy instead of oxidative phosphorylation, even in a sufficient oxygen state. This phenomenon is called “Warburg effect” (Warburg, 1956). On the contrary, normal cells produce ATP mainly through oxidative phosphorylation (Figure 3). One glucose molecule can synthesize 38 ATP molecules through oxidative phosphorylation, while synthesis of 2 ATP molecules through aerobic glycolysis (Feng et al., 2020). Although aerobic glycolysis synthesizes far less energy than oxidative phosphorylation, aerobic glycolysis synthesizes a large number of intermediates necessary for anabolism. Aerobic glycolysis has an important effect on tumor metabolic pathways, tumor microenvironment, and signaling pathways in tumors. Therefore, it is of great significance to explore the mechanisms of aerobic glycolysis in tumorigenesis and tumor progression, which may provide new therapeutic targets or strategies in tumor treatment.

### 3.1 Aerobic glycolysis laid the foundation of proliferation in tumors

Tumorigenesis is a relatively long-term and steady pathological process, phenotypically characterized by uncontrolled cell proliferation (Coleman and Parlo, 2021). Altered metabolisms, which are one of the critical hallmarks of cancer, provide tumor cells with the necessary energy and structural resources for rapid proliferation (Shuvalov et al., 2021). Besides, the large number of intermediates produced during aerobic glycolysis provides the necessary substrates for the rapid and sustained proliferation of tumor cells (Vaupel et al., 2019). Furthermore, compared with oxidative phosphorylation, aerobic glycolysis produces fewer reactive oxygen species to circumvent the deleterious effects of cancer cells. Numerous studies have documented that aerobic glycolysis is pivotal to support the rapid proliferation of malignant cells. The glycolytic enzyme Enolase 1 (ENO1) promotes serine/threonine kinase (AKT) activation to exert its metabolic effects, and the AKT/mTOR signaling pathway is essential for glucose metabolism. It is quite a coincidence that circRPN2 could bind to ENO1 and accelerates its degradation to promote glycolytic reprogramming through the AKT/mTOR pathway, thereby inhibiting hepatocellular carcinoma (HCC) proliferation and metastasis (Li et al., 2022). This indicates that aerobic glycolysis is closely related to tumor proliferation. Besides, pyruvate dehydrogenase kinase 1 (PDK1) was demonstrated to be overexpressed in non-small cell lung cancer tissues and promote cell proliferation and migration by modulating aerobic glycolysis (Liu and Yin, 2017). Similarly, the expression of pyruvate kinase M2 (PKM2) and glucose transporter1 (GLUT1), two important enzymes in aerobic glycolysis, was upregulated by Lin28A/SNHG14/IRF6 axis, thereby reprogramed glucose metabolism and stimulate cell proliferation in glioma cells (Lu et al., 2020). Hexokinase3 (HK3) is a key gene in aerobic glycolysis and participates in the first step of aerobic glycolysis (Kudryavtseva et al., 2016), it could promote the rapid proliferation, invasion, and metastasis of clear cell renal cancer cells by suppressing apoptosis and enhancing epithelial-mesenchymal transition (EMT) (Zhang et al., 2021).

In summary, aerobic glycolysis plays a necessary role in tumor proliferation. In contrast, inhibition of aerobic glycolysis maybe a promising method to suppress tumor growth and proliferation. For example, miR-30a-5p inhibits breast cancer cell proliferation, invasion, and metastasis both *in vitro* and *in vivo* by dampening aerobic glycolysis (Li et al., 2017).

### 3.2 Aerobic glycolysis laid the foundation of invasion and metastasis in tumors

Invasion and metastasis are the leading causes of cancer death (Romano et al., 2014). The ratio between glycolytic and oxidative ATP flux rate is potently associated with cancer invasion and metastasis behavior (Yizhak et al., 2014). Aerobic glycolysis provides nicotinamide adenine dinucleotide phosphate (NADPH) and ATP, as well as the carbon skeletons and intermediates obtained from its high rate of glucose fermentation, as components of biomolecular synthesis that are used by cancer cells to support their rapid growth and metastasis, invasiveness, and chemoresistance (Mullarky and Cantley, 2015; Abdel-Wahab et al., 2019). Unsurprisingly, a recent study showed that glycolysis is the major source of ATP production in endothelial cells and that the silencing of the glycolytic regulator 6-phosphofructo-2-kinase/fructose-2,6-biphosphatase 3 (PFKFB3) impairs the cell metastasis capacity and interferes with vessel sprouting (De Bock et al., 2013). In addition, the expression changes of rate-limiting enzymes in aerobic glycolytic can greatly affect cancer metastasis. For example, the glycolytic enzyme ENO2 promotes the growth and invasion of clear cell renal cancer cells through aerobic glycolysis (Zhang et al., 2021). Hexokinase (HK) is the first rate-limiting enzyme in aerobic glycolytic. It has been reported that the increased expression of HK3 is related to EMT in colorectal cancer, which is involved in the rapid growth and metastasis of colorectal cancer (Pudova et al., 2018). Moreover, the abnormally high expression of fructose-bisphosphatase 1 (FBP1), a downstream glycolysis enzyme and tumor suppressor which was recognized as a glycolysis inhibitor inhibits the invasion and metastasis of breast cancer (Shi et al., 2017). In contrast, the low expression of FBP1 promoted hepatocellular carcinoma cells metastasis through aerobic glycolysis (Yang et al., 2017). These enzymes accelerate the rate of glycolysis, producing faster and more ATP.

Interestingly, the generation of ATP and intermediates also promotes the generation of toxic levels of lactate (Doherty et al., 2014), which aids cancer metastasis and invasion mainly by causing an acidic environment. Many studies have found that a higher lactate level accompanied by enhanced aerobic glycolytic is significantly correlated with recurrence and high metastatic potential of tumors resulting in poor patient outcomes (Walenta et al., 2000). Another study reveals that lactate increases the uptake of folate and glucose, and further increases breast cancer cell T47D metastasis capacity (Guedes et al., 2016).

Consequently, the demand for developing drugs that could attenuate metastasis of malignancies *via* aerobic glycolysis is highly appreciated. Yi et al. reported in 2019 that betulinic acid, a pentacyclic triterpene widely found in birch bark extracts, could restrain breast cancer invasion and metastasis by inhibiting aerobic glycolysis through GRP78/PERK/β-catenin/c-Myc signaling pathway (Zheng et al., 2019b). Collectively, these findings provide a further prospect that the study of aerobic glycolysis inhibition is a promising new strategy for anti-invasion and anti-metastasis in malignancies.

### 3.3 Aerobic glycolysis laid the foundation of epithelial-mesenchymal transition in tumors

The initiation of metastasis is closely associated with EMT (Ribatti et al., 2020). EMT is a process in which epithelial cells lose their intercellular adherence and cellular polarity and acquire the mesenchymal phenotype. EMT is a crucial process in embryogenesis, organ fibrosis, and cancer metastasis (Greenburg and Hay, 1982). Recently, it has become apparent that EMT is tightly associated with aerobic glycolysis. Induction of EMT is associated with heightened rates of glycolysis and lactate production (Liu et al., 2016). Several studies have demonstrated that the master-regulators of EMT transcriptional factors such as zinc finger E-box binding homeobox 1 (Zeb1), snail zinc finger protein (Snail), and twist protein (Twist) are also able to regulate metabolic modulations (Georgakopoulos-Soares et al., 2020). Zeb1 was shown to induce aerobic glycolysis in pancreatic cancer cell models by repressing mitochondrial-localized tumor suppressor sirtuin 3 (SIRT3) (Krebs et al., 2017). Twist was shown to enhance glucose consumption and lactate production. Yang et al. (2015) found that Twist augments PKM2, Lactate dehydrogenase A (LDHA), and glucose-6-phosphate dehydrogenase (G6PD) by activating β1-integrin/FAK/PI3K/AKT/mTOR axis in MCF10A mammary epithelial cells. Similarly, Snail also promotes the metastatic spread by affecting glucose metabolism. Kim and his colleagues showed that Snail reprograms glucose metabolism by repressing phosphofructokinase (PFKP) which switches the glucose flux towards the pentose phosphate pathway in breast cancer (Jo et al., 2020). Besides, Snail-mediated increase in glucose uptake and lactate production was also demonstrated in gastric cancer. Snail-FBP1 signaling axis serves as an effective therapeutic target for primary tumor EMT and glucose metabolism reprogramming (Yu et al., 2017). Consequently, inhibiting aerobic glycolysis maybe a new avenue for suppressing EMT in cancer.

### 3.4 Aerobic glycolysis laid the foundation of apoptosis in tumors

The orderly and delicate regulation of apoptosis under physiological and pathological conditions is an autonomous clearance mechanism adopted by cells to maintain their homeostasis (Galluzzi et al., 2018). However, insufficient apoptosis induces neoplastic diseases, such as cancer. Increasing evidence indicates the close link between aerobic glycolysis and apoptosis resistance in tumor progression as well as poor patient outcomes (Gu et al., 2017). Metabolism can directly or indirectly regulate the apoptotic machinery, cancer cells regulate aerobic glycolysis to escape apoptosis (Matsuura et al., 2016). Lactate acid, the end product of aerobic glycolysis, induces the expression of glycolytic enzymes PFK1 in tumor cells, enhance the supply of ATP and resist cellular apoptosis. PFK15, a glycolytic inhibitor, could rapidly reduce the glucose uptake and induce apoptosis of lung carcinomas cancer cells both *in vitro* and vivo (Clem et al., 2013). Similarly, S100A10 activated mTOR pathway by interacting with annexin A2 to accelerate tumor aerobic glycolysis, promoted malignant proliferation, and suppressed cell apoptosis in gastric cancer (Li et al., 2020b).

In summary, aerobic glycolysis could affect cancer cell apoptosis, and aerobic glycolysis inhibition maybe a novel way to promote cancer cell apoptosis. Such as, several HK inhibitors, including the catalytic inhibitors 3-Bromopyruvate (3-BrPyr), Lonidamine, and the glucose-analogue, competitive inhibitor 2-Deoxyglucose (2-DG) both target HK2 in many tumor models, detach it from mitochondria and elicit tumor cell death (Garcia et al., 2019).

### 3.5 Aerobic glycolysis maintains the stemness property of cancer stem cells

Cancer stem cells are a rare subpopulation of cells that exhibit self-renewal properties and higher tumorigenicity compared with normal tumor cells (Magee et al., 2012). They are recognized as driving forces behind tumor growth (Mamouni et al., 2021). Emerging evidence indicates that metabolic reprogramming, especially the shift of glucose metabolism from mitochondrial oxidative phosphorylation to aerobic glycolysis which is known as Warburg effect, is a prerequisite step for the generation of the cancer stem cells, modulates the phenotype of cancer stem cells, and reshapes the tumor microenvironment (Chen et al., 2019a; Thakur and Chen, 2019). Recently, some studies revealed that aerobic glycolysis is tightly associated with the stemness property of cancer stem cells. And the product of aerobic glycolysis lactate further enhanced the stemness properties of cancer stem cells. Malignancies are recognized as a kind of devastating disease characterized by persistent hypoxia. Hypoxia-inducible factor-2α (HIF-2α), which is critical for tumor cells to adapt to the hypoxic microenvironment, enhances tumor stemness by elevating the expression level of stemness-associated transcriptional factors Nanog and Oct4 through classic Wnt/β-catenin signaling pathway (Zhang et al., 2017). β-arrestin1 (ARRB1) regulated the metabolic preference of bladder cancer stem cells and functioned as a molecular switch which promoted reprogramming towards glycolysis by negatively regulating mitochondrial pyruvate carrier 1 (MPC1) and positively regulating GLUT1 along with glucose uptake (Mamouni et al., 2021). The glycolytic enzyme ENO1 can improve the stemness of gastric cancer stem cells by prominently enhancing the cell’s aerobic glycolysis (Yang et al., 2020). Besides, accumulating research have shown that chemoresistance of cancer stem cell results from dysregulation of glucose metabolism. Tao et al. demonstrated the chemoresistance of gemcitabine (GEM) in pancreatic cancer treatment is due to its metabolic reprogramming and cancer cell stemness enhancement. GEM treatment induced a metabolic shift from mitochondrial oxidation to aerobic glycolysis, which further promoted cancer cell stemness through KRAS/AMPK signaling (Zhao et al., 2019).

Interestingly, lncRNAs can regulate the stemness of cancer stem cells by regulating aerobic glycolysis. LncRNAs described in EMT regulation or metabolic reprogramming are freshly discovered to contribute to CSC creation and stemness maintenance by interacting with self-renewal transcriptional factors such as Nanog homeobox (NANOG), organic cation/carnitine transporter4 (OCT4) and SRY-box transcription factor 2 (SOX2). For example, lncRNA NEAT1 promoted glycolysis by regulating miRNAs (Tan et al., 2020). In addition, NEAT1 regulated CSC properties including self-renew and chemo-resistance in triple-negative breast cancer and lung cancer stem cells by upregulating the expression of pluripotency regulators SOX2, CD44 molecule (CD44), and aldehyde dehydrogenase (ALDH) in a Wnt signaling dependent manner (Shin et al., 2019). Therefore, aerobic glycolysis is closely related to the stemness property of cancer stem cells, and lncRNA is emerging as a pivotal regulator of aerobic glycolysis in CSC, but its overall role in CSC reprogramming needs to be further explored in future studies.

### 3.6 Aerobic glycolysis is closely associated with tumor immune microenvironment

Tumor immune microenvironment (TIME) refers to distinct populations of innate and adaptive immune cells, accompanied by different degrees and types of immune cell infiltration (Togo et al., 2020), including mast cells, macrophages, neutrophils, myeloid-derived suppressor cells (MDSCs), B cells, CD4^+^ T helper (Th) cells, regulatory T cells (Tregs), CD8^+^ cytotoxic T lymphocytes (CTLs), natural killer (NK) cells, dendritic cells (DCs) and some other innate immune cells. TIME influences the immune escape of cancer, and the response and outcome of immunotherapy (Zhang et al., 2020c). The immune response is related to the dramatic modifications in tissue metabolism, including the depletion of nutrients, the increase of oxygen consumption, and the production of reactive nitrogen and oxygen intermediates (Terry et al., 2020). Cancer cells suppress anti-tumor immune response by competing for nutrients or depleting nutrients or reducing the metabolic fitness of tumor-infiltrating immune cells in TIME (Guerra et al., 2020). Moreover, metabolites in TIME also influence immune cells differentiation and effector function. In recent years, the intimate relationship between multifaceted alterations in tumor metabolism and their subsequent influence on immune regulation has become increasingly recognized as an important factor contributing to tumorigenesis and tumor progression (Kesarwani et al., 2017). As we all know, tumor cells generate ATP mainly through aerobic glycolysis, subsequently promoting their proliferation (Altman et al., 2016). However, a new study described that enhanced aerobic glycolysis not only supports the proliferation of cancer cells but also supports the bioenergetic and biosynthetic needs of immune cells (Pearce and Pearce, 2013). Moreover, several clinical studies indicated that aerobic glycolytic activity in human tumors is negatively associated with host antitumor immune responses and the prognosis of anticancer immunotherapy (Jia et al., 2020). Furthermore, some rate-limiting enzymes in the glycolytic pathway act as hallmarks of malignancies, such as HK2 and PKM2, which are responsible for the regulation of immune evasion (Feng et al., 2020).

To date, many studies revealed some immune cells in TIME, such as T cells, and TAMs, which could affect tumorigenesis and tumor progression linked to aerobic glycolysis. T cells play an important role in anti-tumor defense because they exert a powerful immunogens-specific response against cancer cells (Guerra et al., 2020). The emerging evidence indicated that the metabolic status of T cells is crucial for their anti-tumor functions through aerobic glycolysis, tumor cells release lactate into the TIME, which interferes with T cell survival and activation (Brand et al., 2016), reversely, TIME has a significant impact on T cell metabolism, differentiation, and function. A study revealed that excessive glucose uptake by tumor cells restricts the anti-cancer activity of tumor-infiltrating T cells, which leads to increased glycolytic capacity, dampened the mechanistic target of rapamycin kinase (mTOR) activity, and allows tumor progression (Chang et al., 2015). LDHA could catalyze pyruvate to lactate in tumor cells through aerobic glycolysis, knockdown LDHA in tumor cells neutralized TIME acidity, and promoted tumor infiltration by CD8^+^ T cells and NK cells while decreasing the number of immunosuppressive immune cells (Zhang et al., 2019). TAMs are the major immunosuppressive components in TIME, accounting for a large proportion of the tumor mass, in addition, they are highly glycolytic and produce large amounts of lactate. A study demonstrated that mTOR signaling is closely connected with the polarization of TAMs from anti-tumoral M1Ф to pro-tumoral M2Ф, anti-tumoral M1Ф regulates aerobic glycolysis in cancer cells leading to reduced proliferation and decreased production of lactate, and lactate was the potent immunosuppressor and angiogenesis stimulator in the tumor microenvironment (Chen et al., 2020a). Furthermore, genetic deletion of LDHA, 2-DG administration, or mTORC1 inhibition has been proposed as therapeutic avenues designed to decrease glycolytic metabolism in cancer cells, reduce lactate in the TIME, and repolarize TAMs to a pro-inflammatory state (Vitale et al., 2019). Thus, affecting aerobic glycolysis might not only improve immune cell responses against highly immunogenetic cancers but also increase the immunogenicity of cancer cells, and targeting aerobic glycolysis might be a novel insight to regulating tumor immune microenvironment, finally inhibit the immune escape of cancer, tumorigenesis, and tumor progression.

## 4 Long non-coding RNAs regulate tumorigenesis and tumor progression through glycolytic enzymes and glucose transporter

The “Warburg effect” has been discovered nearly a hundred years ago, however, there are still many mysteries about the molecular mechanisms of aerobic glycolysis in tumors, especially the roles of lncRNAs in the regulation of aerobic glycolysis. Recent studies have implicated the roles of lncRNAs in the regulation of glycolysis. Some lncRNAs are reported to modulate the expression levels of glycolytic enzymes (Fan et al., 2017). Some lncRNAs are known to regulate glycolysis-related transcription factors or signaling pathways. For example, lincRNA-p21 (Yang et al., 2014), linc-AC020978 (Hua et al., 2020), and LINK-A (Lin et al., 2016), have been reported to promote glycolysis under hypoxic conditions through HIF1α. The lncRNA-MEG3 can depress aerobic glycolysis *via* p53 and functions as a tumor suppressor in liver cancer cells (Zhang et al., 2003; Zhou et al., 2007). These studies indicated that lncRNAs may play an important role in glycolysis, but the biological functions and underlying mechanisms should be evaluated in-depth. Particularly, there is no evidence confirming that lncRNAs regulate the balance between aerobic glycolysis and oxidative phosphorylation by directly regulating related signaling pathways. Here, we reviewed that lncRNAs could regulate tumorigenesis and tumor progression through glycolytic enzymes and glucose transporter.

### 4.1 Long non-coding RNAs regulate tumor metabolism and tumorigenesis, and tumor progression through the enzymes of aerobic glycolysis

#### 4.1.1 Long non-coding RNAs regulate tumor metabolism and tumorigenesis, and tumor progression through pyruvate kinase M2

**FIGURE 4:**
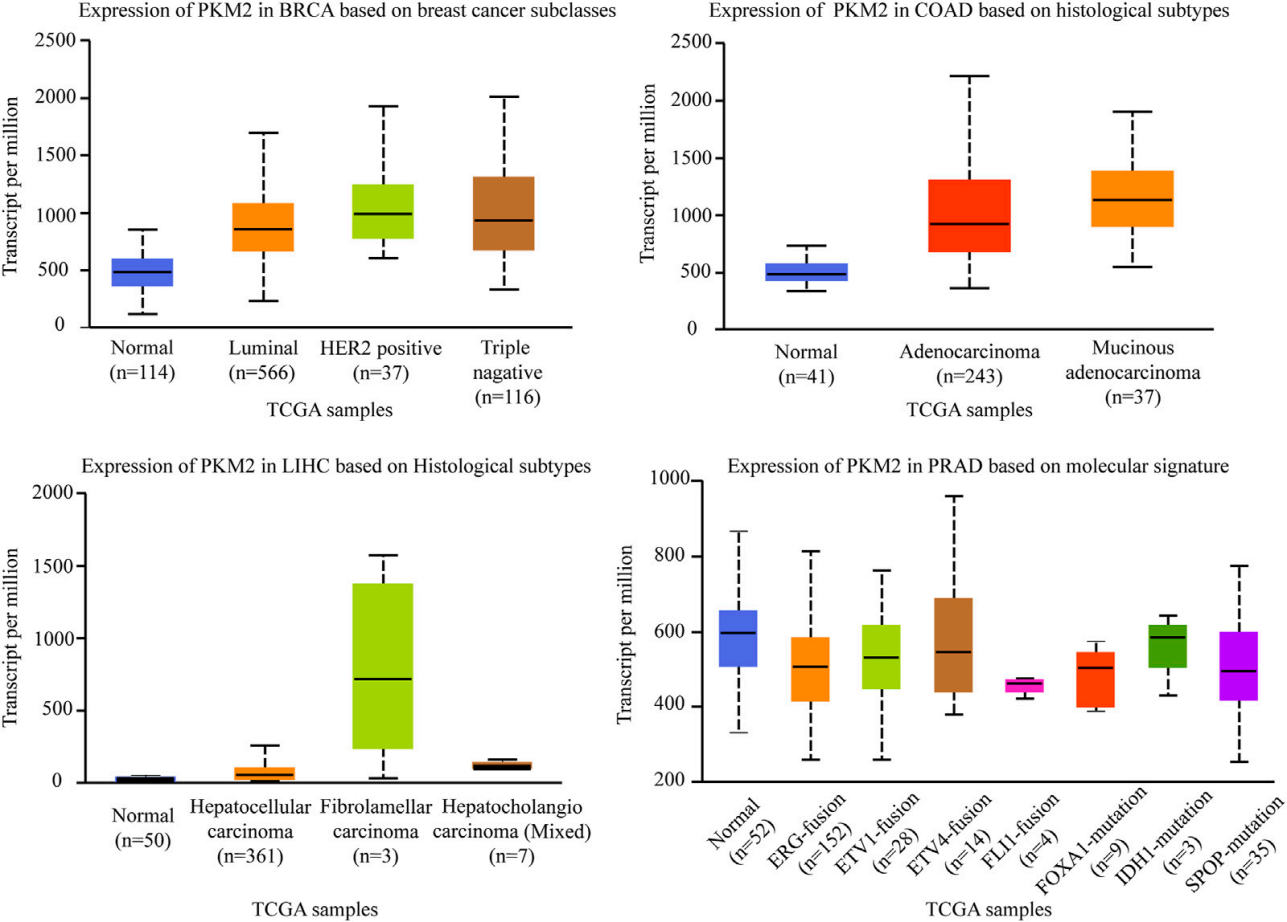
Association of PKM2 gene expression with histological subtypes and molecular signature in different cancers. BRCA, breast invasive carcinoma. COAD, colon adenocarcinoma. LIHC, liver hepatocellular carcinoma. PRAD, prostate adenocarcinoma.

Pyruvate Kinase (PK) is a class of known glycolytic enzymes involved in the last step of glycolysis by converting phosphoenolpyruvate (PEP) to pyruvate. Four isoforms of pyruvate kinase have been identified: PKM1, PKM2, PKR, and PKL (Chen et al., 2020b). PKL and PKR, which are encoded by the PKLR gene, are expressed in some special tissues, such as liver, kidney, and erythrocytes (Massari et al., 2016), respectively, whereas PKM1 and PKM2, are encoded by the same PKM gene *via* alternative RNA splicing (Taniguchi et al., 2020). Compared to the other three subtypes, PKM2 is an embryonic isoform that is highly expressed in embryos with lower enzymatic activity than PKM1 (Christofk et al., 2008a). During embryogenesis, PKM2 is progressively replaced by the other three isoenzymes. Conversely, during tumorigenesis, PKM2 gradually replace the other three subtypes and showed a tendency of high expression in tumor tissues (Mazurek, 2011). For example, there is a switch from the PKM1 isoform to the PKM2 isoform in various cancers like glioblastoma and hepatocellular carcinoma, which enhances the level of aerobic glycolysis in tumors and promotes tumor formation and proliferation (Christofk et al., 2008b). Therefore, suppressing PKM2 is significantly important for inhibiting carcinogenesis and the development of carcinomas. PKM2pS37 is the best-studied form of PKM2 phosphorylation to date and triple-negative breast cancer is the most aggressive breast cancer subtype. A study demonstrated that PKM2pS37, as the prognostic indicator of triple negative breast cancer outcomes, has the potential to impact triple-negative breast cancer patients directly with CDK inhibitors and pyruvate kinase activators alone or in combination (Apostolidi et al., 2021). Consistent with these studies, we defined that the expression level of PKM2 is significantly different in four subtypes of breast cancer. As shown in Figure 4, PKM2 is relatively highly expressed in luminal, HER2^+^, and triple-negative breast cancers in the different subclasses of breast cancers. It is not the only case, in the different histological subtypes of colon adenocarcinomas, PKM2 is also relatively highly expressed in adenocarcinoma and mucinous adenocarcinoma. In the different histological subtypes of liver hepatocellular carcinomas, PKM2 especially showed the highest expression level in fibrolamellar carcinoma. However, although PKM2 showed relatively low expression levels in the ERG fusion, FOXA1 mutation, and SPOP mutation molecular signature of prostate adenocarcinomas, there were no significant differences in several other molecular signatures of prostate adenocarcinoma (The data comes from UALCAN).

According to recent studies, lncRNAs are involved in tumor metabolism and proliferation by regulating the enzymatic or transcriptional activity of PKM2 and related signaling pathways. LncRNA-FEZF1-AS1 could enhance aerobic glycolysis, promote proliferation and metastasis of colorectal cancer, and increase the stability and expression level of PKM2 in cytoplasmic and nucleus. And the increasing expression of PKM2 in cytoplasm promoted lactate production in colorectal cancer cells (Bian et al., 2018). LncRNA-WFDC21P markedly inhibited hepatocellular carcinoma cell’s proliferation and metastasis *via* modulating the process of glycolysis by binding to PKM2 and suppressed its transcriptional activity, which inhibited the activity and nuclear translocation of HIF1α (Guan et al., 2020). LncRNA-HOXB-AS3 peptide suppressed colon cancer (CRC) growth through blocking hnRNPA1-mediated PKM splices, thereby inhibiting the formation of PKM2 and suppressing the reprogramming of the glucose metabolism (Huang et al., 2017). Several lncRNAs have been found to investigate their roles in liver cancer development and progression. Linc01554 downregulation empowers HCC cancer cells to acquire high aerobic glycolysis and sustain cells’ growth advantages. Zheng YL et. demonstrated that Linc01554 plays a tumor suppressor role in regulating ubiquitin-mediated degradation of PKM2 and inhibiting Akt/mTOR signaling pathway (Zheng et al., 2019a). LncRNA-SOX2OT promotes HCC metastasis by upregulating PKM2, which increases the glycolytic pathway in HCC cells and thereby enhances EMT (Liang et al., 2020). Thus, PKM2 may be closely related to lncRNAs-mediated tumor pathogenesis and development. However, further studies are needed to explore the underlying molecular mechanism.

#### 4.1.2 Long non-coding RNAs regulate tumor metabolism and tumorigenesis, tumor progression through lactate dehydrogenase A

**FIGURE 5:**
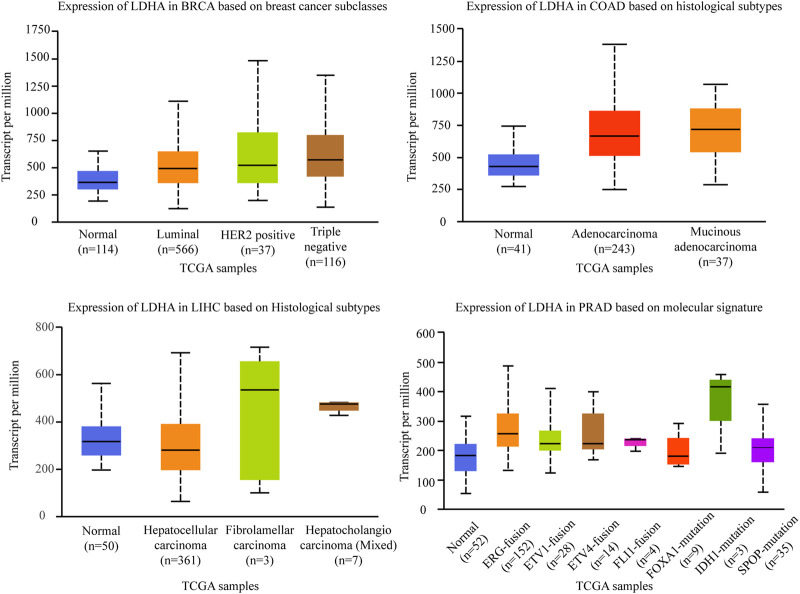
Association of LDHA gene expression with histological subtypes and molecular signature in different cancers. BRCA, breast invasive carcinoma. COAD, colon adenocarcinoma. LIHC, liver hepatocellular carcinoma. PRAD, prostate adenocarcinoma.

Lactate dehydrogenase (LDH) comes from a family of NAD^+^-dependent enzymes, which act as a tetrameric enzyme with distinct catalytic activity. The LDH family comprises two major subunits: A and/or B, resulting in five major isozymes: A4, A3B1, A2B2, A1B3, and B4. LDH catalyzes the conversion of pyruvate to lactate and NADPH to Nicotinamide adenine dinucleotide (NADH) and produces energy in the form of ATP, which is the end product of glycolysis. Lactate dehydrogenase A (LDHA), also known as LDH-5, M-LDH, or A4, which is the predominant form in skeletal muscle, kinetically prefers the converting pyruvate to lactate in aerobic glycolysis in tumors. The overexpression of LDHA has been established in various solid cancers, including renal, pancreatic, non-small cell lung cancer, colorectal cancer, breast cancer, and other gynecologic cancers (Goldman et al., 1964). Molecular mechanism studies indicated that LDHA plays critical roles in tumor maintenance and aggravation, including promoting cancer cell proliferation, epithelial to mesenchymal transition (Jiang et al., 2016), angiogenesis (Giatromanolaki et al., 2006), cytoskeletal remodeling (Arseneault et al., 2013), cell motility, invasion and metastasis (Liu et al., 2015). Correspondingly, LDHA inhibition is shown to impair tumorigenesis and tumor growth (Le et al., 2010; Baig et al., 2019). Besides, it has been observed in several studies that inhibition of LDHA causes no significant toxic effect on normal tissue, which makes LDHA a promising therapeutic target in cancer (Tachtsidis et al., 2016). Such as, inhibiting LDHA with FX11 (LDHA inhibitor) suppressed pyruvate to lactate conversion, and caused reductions in ATP levels and substantial oxidative stress in cancer cells (Le et al., 2010). We evaluated the gene expression level of LDHA within different molecular or histological subtypes in four cancer types in TCGA publications. As shown in Figure 5, the gene expression analyzed are: 1) LDHA is relatively highly expressed in luminal, HER2^+^, and triple-negative breast cancers in the different subclasses of breast cancers. 2) In different histological subtypes of colon adenocarcinomas, LDHA is also relatively highly expressed in adenocarcinoma and mucinous adenocarcinoma. 3) Similarly, in the different histological subtypes of liver hepatocellular carcinomas, LDHA is relatively highly expressed in hepatocholangio carcinoma (Mixed). 4) Similar result was also found in prostate adenocarcinoma, LDHA is especially highly expressed in ERG fusion, ETV1 fusion, ETV4 fusion, IDH1 mutation, and SPOP mutation subtype, there were no significant differences in several other molecular signatures of prostate adenocarcinomas such as FLI1 fusion and FOXA1 mutation (The data comes from UALCAN).

Recent studies have focused on the regulatory function of lncRNAs in cancer cell glucose metabolism through LDHA. LncRNA-IGFBP4-1 has been demonstrated to increase ATP production in lung cancer cells by upregulating metabolism enzymes expression, including HK2, LDHA, and PDK1 (Yang et al., 2017a). Wang et al. (2020a) revealed that lncRNA HULC orchestrates enzymatic activities of two glycolytic enzymes, LDHA and PKM2, by directly adapting them to binding to fibroblast growth factor receptor type 1 (FGFR1), and eventually promoting Warburg effect in hepatocellular carcinoma cells. Over-activated metabolic signaling pathways were found to be involved in lncRNAs related to rapid ATP production and proliferation in cancer cells. For example, lncRNA ANRIL up-regulated the expression of Glut1 and LDHA by promoting the phosphorylation of Akt to activate the mTOR signal pathway (Zou et al., 2016). LncRNA RAET1K upregulated the expression of LDHA, decreased the expression of miR-100-5p, then upregulated the ICMT-Rac1 signaling pathway, thus promoting HCC metastasis (Zhou et al., 2020). Thus, LDHA may be another target that is related to lncRNAs and mediated tumor pathogenesis and development, but the underlying mechanism needs to be urgently investigated.

#### 4.1.3 Long non-coding RNAs regulate tumor metabolism and tumorigenesis, tumor progression through Hexokinase2

**FIGURE 6:**
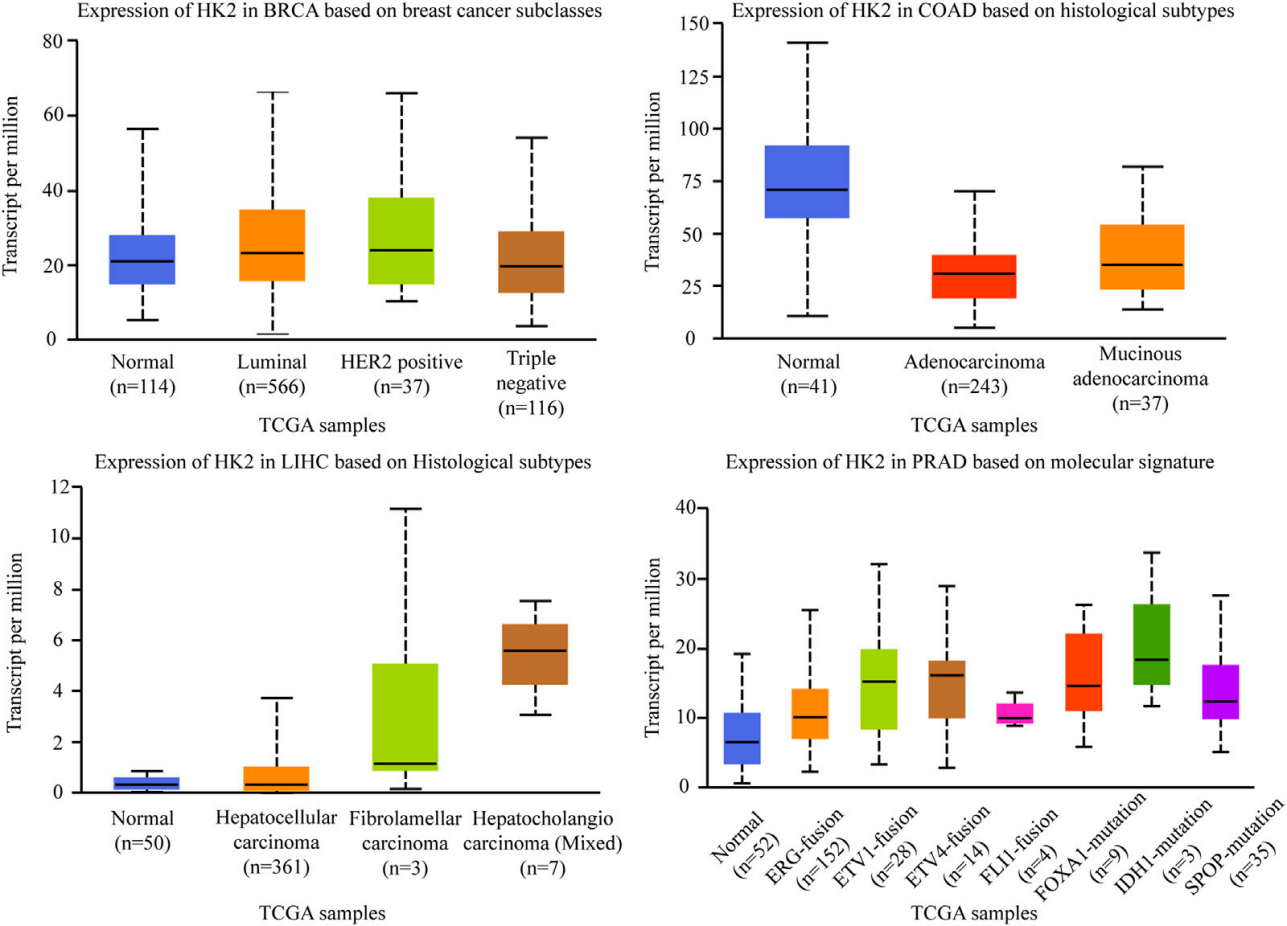
Association of HK2 gene expression with histological subtypes and molecular signature in different cancers. BRCA, breast invasive carcinoma. COAD, colon adenocarcinoma. LIHC, liver hepatocellular carcinoma. PRAD, prostate adenocarcinoma.

Hexokinase (HK) is a group of rate-limiting enzymes in the glycolysis pathway, which using ATP as a phosphate donor, catalyzes the phosphorylation of glucose to produce glucose 6-phosphate (Lu and Hunter, 2018). There are four isoforms of HK: HK-Ⅰ, HK-Ⅱ, HK-Ⅲ, and HK-Ⅳ. HK-Ⅰ, and HK-Ⅲ are ubiquitously expressed, while the expression of HK-Ⅳ, glucokinase, is restricted primarily to the liver and pancreas. Different from these three isoforms, HK-Ⅱ (HK2) is the predominant isoform in insulin-sensitive tissues such as adipose, skeletal, and cardiac muscle. HK2 catalyzes the first committed step of glucose metabolism and initiates the major pathways of glucose utilization, it is confirmed to be a tumor promoter and plays an important regulating role in glucose metabolism in multiple malignancies, including breast cancer, lung cancer, and liver cancers (Kawai et al., 2005; Zhang et al., 2020a). Besides, HK2 has been recognized to regulate the malignant phenotype of cancer cells, such as cellular apoptosis and migratory capabilities (Lis et al., 2016; Chen J. et al., 2019c; Feng et al., 2020). Among the different subtypes of cancers, the expression of HK2 is diverse. As shown in Figure 6, there was no significant correlation between HK2 expression and molecular or histological subtypes in breast cancers. In the different molecular of colon adenocarcinomas, HK2 is relatively lowly expressed in adenocarcinoma and mucinous adenocarcinoma. While in the different histological subtypes of liver hepatocellular carcinomas, HK2 is relatively highly expressed in hepatocellular carcinoma. A similar result was also found in prostate adenocarcinoma, HK2 is especially highly expressed in ERG fusion, ETV1 fusion, ETV4 fusion, FOXA1 mutation, IDH1 mutation, and SPOP mutation subtype, there was no significant difference in FLI1 fusion (The data comes from UALCAN).

Recently, there are several studies revealed that lncRNAs can regulate tumor metabolism and proliferation through HK2. Some studies indicated that lncRNA could act as a sponge of miRNA, enhance the expression of HK2, and promote cancer cell proliferation, metastasis, and invasion. For example, lncRNA PVT1 could act as a molecular sponge of miR-497, promote the expression of HK2, enhance the uptake of glucose and production of lactate, and promote osteosarcoma cell proliferation (Song et al., 2017). LncRNA C1QTNF1 could sponge miR-484 and consequently increase HK2 expression, promoting colorectal cancer cell proliferation, migration, and invasion (Jin et al., 2020a). LncRNA DUXAP8 could directly sponge miR-409-3p to regulate HK2 expression, and promote non-small-cell lung cancer cell growth, and metastasis (Yin et al., 2020). LncRNA HOTTIP promoted glycolysis under hypoxia by directly binding to miR-615-3p, acting as a molecular sponge, thus regulating the protein expression of hexokinase 2 (HK2) and high mobility group box 3 (HMGB3) (Shi et al., 2019). Interestingly, HOTTIP has also been demonstrated to stimulate CSC properties by mediating the activation of stemness and self-renewal transcriptional factors NANOG, OCT4, and SOX2 through Wnt/β-catenin signaling pathway (Han et al., 2020). Some studies indicated that lncRNA could regulate signaling pathways, and affect aerobic glycolysis and tumorigenesis. For example, lncRNA UCA1 could activate the mTOR pathway, mediate the regulation of urothelial cancer associated 1 (UCA1) to HK2 through activation of signal transducer and activator of transcription 3 (STAT3), then promote glycolysis, exert promotion effects on tumorigenesis in bladder cancer (Li et al., 2014). LncRNA BCAR4 coordinated the Hedgehog signaling pathway to enhance the transcription of glycolysis activators HK2, facilitating tumorigenesis in breast cancer (Zheng et al., 2017). Accordingly, HK2 may be related to lncRNA and co-regulated tumor pathogenesis and development, but the underlying mechanism needed to be further studied.

### 4.2 Long non-coding RNAs regulate tumor metabolism and tumorigenesis, tumor progression through glucose transporters1

Tumor cells have an increased dependence on extracellular glucose, thus glucose transporters (GLUTs) constitute also an anticancer target (Barbosa and Martel, 2020). The GLUT family is facultative transporter that play a vital role in glucose transport across the plasma membrane, which is the initial step of glycolysis. The family of transporters is composed of 14 members: GLUT1-GLUT12, GLUT14, and the H^+^/myo-inositol transporter. Each of the GLUT transport proteins possesses different affinities for glucose. Among these, GLUT1, GLUT3, and GLUT4 have a high affinity for glucose, allowing the transport of glucose at a higher rate under normal physiological conditions (Mueckler and Thorens, 2013). GLUT2 found predominantly in liver, intestine, kidney, and pancreatic β-cells is a low-affinity glucose transport protein that is part of the glucose sensor in pancreatic β-cells and facilitates either glucose uptake or efflux from the liver depending on the nutritional state. GLUT3 is the glucose transporter responsible for maintaining an adequate that is responsible for insulin-regulated glucose disposal. Distinguished from several other glucose transporters, GLUT1 is a facilitative glucose transporter that belongs to the solute-linked carrier gene family SLC2 and is overexpressed ubiquitously in human cancer cells (Ganapathy et al., 2009), including breast, lung, renal, colorectal, and pancreatic cancers (Szablewski, 2013), which has potential effects on glycolysis process in cancer (Shang et al., 2020). Consistent with GLUT1’s overexpression, GLUT1 is crucial for the uptake of glucose by breast cancer cells and is also the main glucose transporter in breast cancer cell lines (Wuest et al., 2018). Besides, the deregulation of GLUT1 is involved in the biological processes of tumor cells, including survival, growth, and metastasis (Goos et al., 2016).

**FIGURE 7:**
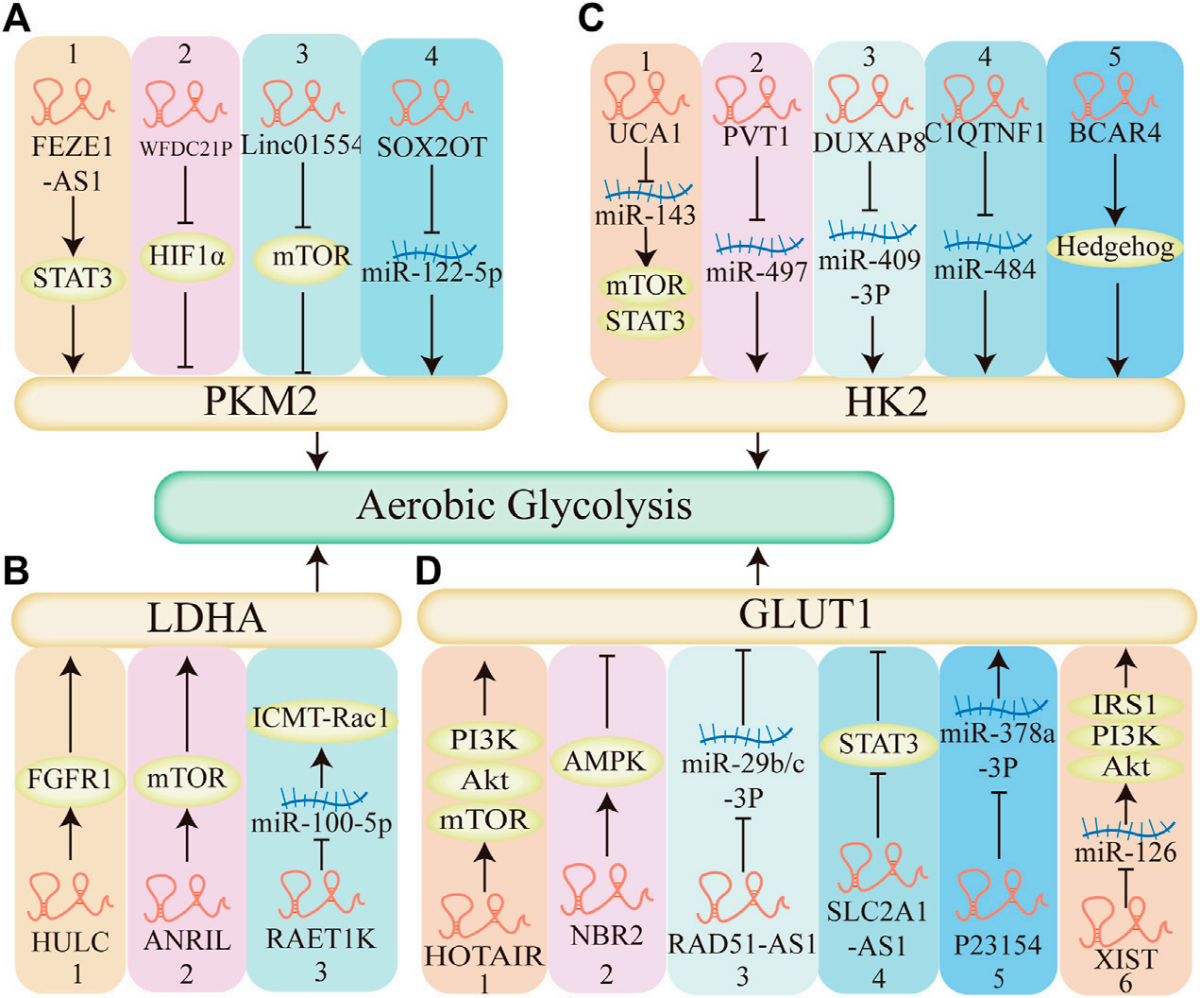
The mechanism of LncRNAs regulate aerobic glycolysis. **(A)** LncRNAs regulate aerobic glycolysis through pyruvate kinase M2 (PKM2). 1–3. LncRNA-FEZF1-AS1, LncRNA-WFDC21P, Linc01554 regulates STAT3, HIF1α, mTOR pathway respectively, affects the expression of PKM2, regulates aerobic glycolysis. 4. LncRNA-SOX2OT decreases the expression of miR-122-5p, upregulates PKM2, promotes aerobic glycolysis. **(B)** LncRNAs regulate aerobic glycolysis through Lactate dehydrogenase A (LDHA). 1–2. LncRNA HULC, LncRNA ANRIL elevates FGFR1 and mTOR, respectively, regulates LDHA, enhances aerobic glycolysis. 3.LncRNA RAET1K decreases the expression of miR-100-5P, upregulates the expression of LDHA, promotes aerobic glycolysis. **(C)** LncRNAs regulate aerobic glycolysis through hexokinase 2 (HK2). 1. LncRNA UCA1 acts as a molecular sponge of miR-143, activates the mTOR pathway, promotes aerobic glycolysis. 5. LncRNA BCAR4 coordinates the Hedgehog signaling pathway, enhances the HK2 and aerobic glycolysis. 2–4. LncRNA PVT1, LncRNA DUXAP8, LncRNA C1QTNF1 acts as a molecular sponge of miR-497, miR-409-3P, miR-484 respectively, promotes the expression of HK2, enhances aerobic glycolysis. **(D)** LncRNAs regulate aerobic glycolysis through glucose transporter1 (GLUT1). 1,2,4: LncRNA HOTAIR, LncRNA NBR2, LncRNA SLC2A1-AS1 regulates mTOR, AMPK, STAT3 pathway respectively and affects the expression of GLUT1, affects aerobic glycolysis. 3,5: LncRNA RAD51-AS1, LncRNA P23154, acts as a molecular sponge of miR-29b/c-3P, miR-378a-3P respectively, regulates the expression of GLUT1, affects aerobic glycolysis. 6. LncRNA XIAT acts as a molecular sponge of miR-126, activates the PI3K pathway and the expression of GLUT1, promotes aerobic glycolysis.

Several studies have shown that lncRNAs can regulate tumor metabolism and proliferation through GLUT1. Some studies indicated that LncRNA could regulate signaling pathways to affect the expression of GLUT1, tumorigenesis, and tumor progression. For example, LncRNA HOTAIR induced GLUT1 expression *via* activating the mTOR pathway, promoting cell proliferation in hepatocellular carcinoma cells and tissues (Wei et al., 2017). LncRNA NBR2 promoted protein kinase AMP-activated catalytic subunit alpha 1 (AMPK) pathway to down-regulate GLUT1 expression and the EMT process, suppressed tumor progression in osteosarcoma cells (Liu and Gan, 2016). LncRNA SLC2A1-AS1 negatively regulated GLUT1 expression then inhibited STAT3 signaling pathway, and markedly decreased the proliferation and metastasis of hepatocellular carcinoma cells (Shang et al., 2020). Some studies indicated that lncRNAs associated with ceRNAs, based on the LncRNA-miRNA-mRNA network to exert their biological functions on the tumorigenesis and tumor progression. For example, Inc-p23154 could inhibit miR-378a-3p transcription, thereby enhancing GLUT1 expression and promoting oral squamous cell carcinoma metastasis (Wang et al., 2018a). LncRNA RAD51-AS1 acted as a sponge of miR-29b/c-3p, which in turn inhibited the expression of GLUT1, ultimately inhibiting proliferation, invasion, and glycolytic metabolism of colorectal cancer cells (Li et al., 2020a). LncRNA XIST functioned as a ceRNA to regulate the IRS1/PI3K/Akt pathway by sponging miR-126, elevated the expression of GLUT1, and promoted glioblastoma cell viability, migration, invasion, and resistance to apoptosis (Cheng et al., 2020) (Table 1; Figure 7). Thus, GLUT1 is also closely related to lncRNA and affects tumor metabolism and proliferation, but the underlying mechanism needed to be further studied.

**TABLE 1:** Overview of LncRNAs related to the enzymes of aerobic glycolysis in cancer.

Enzyme	LncRNAs	Target	Function	Cancer type
Pyruvate kinase M2 (PKM2)	LncRNA-FEZF1-AS1	PKM2/STAT3	Increase the stability and expression level of PKM2 in cytoplasmic and nucleus, promote colorectal cancer cell proliferation and metastasis	Colorectal cancer
	LncRNA-WFDC21P	PKM2/HIF1α	Bind to PKM2 and suppress its transcriptional activity, inhibit hepatocellular carcinoma cell’s proliferation and metastasis	Hepatocellular carcinoma
	LncRNA-HOXB-AS3	hnRNP A1/PKM2	Ensure the formation of lower PKM2, suppress colon cancer growth	Colon cancer
	Linc01554	PI3K/Akt/mTOR	Regulate ubiquitin-mediated degradation of PKM2, suppress hepatocellular carcinoma cancer cell growth	Hepatocellular carcinoma
	LncRNA-SOX2OT	miR-122-5p	Upregulate PKM2, promote hepatocellular carcinoma cancer cell metastasis	Hepatocellular carcinoma
Lactate dehydrogenase A (LDHA)	LncRNA-IGFBP4-1	IGFBP4	Upregulate LDHA expression, promote lung cancer progression	Lung cancer
	LncRNA HULC	FGFR1	Elevate phosphorylation and activity of LDHA, enhance hepatocellular carcinoma cancer cell proliferation	Hepatocellular carcinoma
	LncRNA ANRIL	PI3K/Akt/mTOR	Upregulate the expression of LDHA, promote nasopharyngeal carcinoma progression	Nasopharyngeal carcinoma
	LncRNA RAET1K	miR-100-5p	Upregulate the expression of LDHA, promote hepatocellular carcinoma cell metastasis	Hepatocellular carcinoma
Hexokinase 2 (HK2)	LncRNA PVT1	miR-497	Enhance the expression of HK2, promote osteosarcoma cell proliferation	Osteosarcoma
	LncRNA C1QTNF1	miR-484	Increase HK2 expression, promote colorectal cancer cell proliferation, migration, and invasion	Colorectal cancer
	LncRNA DUXAP8	miR-409-3p	Regulate HK2 expression, promote non-small-cell lung cancer cell growth, metastasis	Non-small-cell lung cancer
	LncRNA UCA1	mTOR-STAT3/miR-143	Mediate the regulation of UCA1 to HK2, exert promotion effects on tumorigenesis in bladder cancer	Bladder cancer
	LncRNA BCAR4	Hedgehog	Enhance the transcription of HK2, facilitate tumorigenesis in breast cancer	Breast cancer
Glucose transporter1 (GLUT1)	LncRNA HOTAIR	PI3K/Akt/mTOR	induce GLUT1 expression, promote hepatocellular carcinoma cell proliferation	Hepatocellular carcinoma
	LncRNA NBR2	AMPK	Down-regulate GLUT-1 expression and the EMT process, suppress tumor progression in osteosarcoma cell	Osteosarcoma
	LncRNA SLC2A1-AS1	STAT3	Negatively regulate GLUT1 expression, decrease the proliferation and metastasis of hepatocellular carcinoma cells	Hepatocellular carcinoma
	Lnc-P23154	miR-378a-3p	Enhance GLUT1 expression and promote oral squamous cell carcinoma metastasis	Oral squamous cell carcinoma
	LncRNA RAD51-AS1	miR-29b/c-3p	Inhibit the expression of GLUT1, inhibit proliferation, invasion and glycolytic metabolism of colorectal cancer cell	Colorectal cancer
	LncRNA XIST	miR-126/IRS1/PI3K/Akt	Elevate the expression of GLUT1, promote glioblastoma cell viability, migration, invasion, and resistance to apoptosis	Glioblastoma

## 5 Long non-coding RNAs serve as a promising target in the treatment of malignancies

To date, the biomarkers are commonly used in early screening for all kinds of cancers (Yuan et al., 2020), and lncRNAs have attracted increasing attention as cancer biomarkers for early screening, diagnosis, prognosis, and responses to drug treatment (Zhang et al., 2018; Necula et al., 2019; Zhuo et al., 2019). LncRNAs constitute an ever-growing category of functional RNA species known to impinge on all hallmarks of cancer (Napoli and Flores, 2020). Many studies revealed that lncRNAs could regulate many important pathological processes in cancer, such as tumorigenesis, tumor progression, proliferation, metastasis, and drug resistance (Fang and Fullwood, 2016; Schmitt and Chang, 2016), suggesting an enormous potential for further development of lncRNA biomarkers in specific cancer histologic analysis. The followings are some evidence that lncRNA can be used as a biomarker and is strongly associated with poor prognosis in breast cancer, colorectal cancer, medulloblastoma, and renal cell carcinoma.

Song E et al. reported that the high expression level of HIFAL, a HIF-1α anti-sense lncRNA, is associated with aggressive breast cancer phenotype and poor prognosis. Mechanistically, HIFAL overexpression promotes tumor progression by forming a positive feed-forward loop with HIF-1α to enhance HIF-1α-mediated transactivation and glycolysis (Zheng et al., 2021). LncRNA TROJAN is highly expressed in Estrogen receptor-positive (ER^+^) breast cancer tissues and promotes cancer proliferation. Thus, lncRNA TROJAN may serve as a potential therapeutic target for ER^+^ breast cancer (Jin et al., 2020b).

LncRNA RAMS11 directly affects colorectal cancer biology, including promoting an aggressive phenotype and correlating with treatment response and resistance, indicating the potential value of lncRNA RAMS11 as a biomarker and therapeutic target for colorectal cancer (Silva-Fisher et al., 2020). LncRNA SNHG11 has been reported as a potential biomarker for the early detection of colon cancer and a new therapeutic target for this disease (Xu et al., 2020).

Lnc-HLX-2-7 is highly upregulated in Group 3 Medulloblastoma (MB) cell lines, promoted cell proliferation and 3D colony formation, inhibited cell apoptosis, indicating that Lnc-HLX-2-7 is oncogenic in MB and represents a promising novel molecular marker and a potential therapeutic target in Group 3 MBs (Katsushima et al., 2021).

LncRNA TRAF31P2-AS1 functions as a tumor suppressor in NONO-TFE3 translocation renal cell carcinoma progression and may serve as a novel target for NONO-TFE3 translocation renal cell carcinoma therapy (Yang et al., 2021).

Therefore, targeting lncRNAs may be a promising strategy to enhance chemosensitivity and improve the efficacy of chemotherapy (Arun et al., 2018). However, there is not enough clinical evidence indicating that lncRNAs could act as biomarkers for the treatment of cancer, more in-depth studies are required to accelerate the clinical applications of lncRNAs.

## 6 Conclusion and prospects

It is now clear that aerobic glycolysis is involved in a range of events important in cancer, including initiation, progression, metastasis, drug resistance, immune evasion, and the dynamic changes in immune microenvironment. As glucose metabolic disarrangement provides substrates for the biosynthesis of biomolecules essential for the rapid development of tumor. And the production of lactate also leads to a lower environmental pH and benefits tumor cell metastasis, invasion, and drug resistance. Moreover, there is ample evidence that high lactate levels have immune-modulatory properties. LncRNAs participate in each of these events by transcriptional, post-transcriptional, and epigenetic gene-regulatory mechanisms.

As master regulators of gene expression, the mis-regulation of lncRNAs expression has been demonstrated to be the driver of tumorigenesis and development associated with metabolic disarrangement. Most commonly, lncRNAs sponge to corresponding miRNA and mRNA that target critical metabolic enzymes, such as PKM2, LDAH, HK2, and glucose transporter GLUT1, to modulate the expression of numerous oncogenes and tumor-suppression genes. Furthermore, lncRNAs also play critical roles in generating an immune-permissive microenvironment by modulating aerobic glycolysis. The reversible transition of EMT and mesenchymal-epithelial transition (MET) is a key event in tumor progression, metastasis, and invasiveness into normal tissues. And the processes of EMT and MET are also highly regulated by lncRNAs. The discovery of lncRNAs in the regulation of “stemness” further broadens its opportunities for the treatment of cancer. Therefore, continued study of lncRNAs in preclinical research will yield new insights into RNA-based therapeutics in cancer.

## Glossary

ATP: Adenosine triphosphate
LncRNAs: Long non-coding RNAs
NcRNAs: Non-coding RNAs
RBPs: RNA binding proteins
TIME: Tumor immune microenvironment
TAMs: Tumor associated macrophages
Tregs: Regulatory T cells
EGFR: Epidermal growth factor receptor
RBM5: RNA-binding motif protein 5
NK cell: Nature killer cell
PPP: Pentose-phosphate pathway
DAPDH: Diaminopimelate dehydrogenase
ENO1: Enolase 1
AKT: AKT serine/threonine kinase
HCC: Hepatocellular carcinoma
PDK1: Pyruvate dehydrogenase kinase 1
GLUT1: Glucose transporter1
HK3: Hexokinase3
EMT: Epithelial-mesenchymal transition
PFKFB3: 6-phosphofructo-2-kinase/fructose-2,6-biphosphatase 3
FBP1: Fructose-bisphosphatase 1
GSH: Antioxidant glutathione
Zeb1: Zinc finger E-box binding homeobox 1
Snail: Snail zinc finger protein
Twist: Twist protein
SIRT3: Sirtuin 3
LDHA: Lactate dehydrogenase A
G6PD: Glucose-6-phosphate dehydrogenase
PFKP: Phosphofructokinase
CSCs: Cancer stem cells
HIF-2α: Hypoxia-inducible factor-2α
ARRB1: β-arrestin1
MPC1: Mitochondrial pyruvate carrier 1
GEM: Gemcitabine
NANOG: Nanog homeobox
OCT4: Organic cation/carnitine transporter4
SOX2: SRY-box transcription factor 2
NEAT1: Nuclear-enrich abundant transcript 1
CD44: CD44 molecule
ALDH: Aldehyde dehydrogenase
ESCs: Embryonic stem cells
MDSCs: Myeloid derived suppressor cells
Th cells: T helper cells
CTLs: CD8^+^ cytotoxic T lymphocytes
DCs: Dendritic cells
TME: Tumor microenvironment
PEP: Phosphoenolpyruvate
CRC: Colon cancer
NADH: Nicotinamide adenine dinucleotide
FGFR1: Fibroblast growth factor receptor type 1
UCA1: Urothelial cancer associated 1
STAT3: Signal transducer and activator of transcription 3
mTOR: Mechanistic target of rapamycin kinase
AMPK: Protein kinase AMP-activated catalytic subunit alpha 1
ER^**+**^: Estrogen receptor-positive
MB: Medulloblastoma
MET: Mesenchymal-epithelial transition
